# Cantharides as a Therapeutical Agent, Internally Administered in Large Doses

**Published:** 1862-06

**Authors:** Alex. McBride

**Affiliations:** Surgeon O.V., U.S.A.


### CANTHARIDES AS A THERAPEUTICAL AGENT, INTERNALLY ADMINISTERED IN LARGE DOSES.

### ITS MODE OF OPERATION.

By ALEX. McBRIDE, M.D., SURGEON O.V., U.S.A.

The profession do not seem to be aware that they have in cantharides an agent the most powerful to rekindle the waning spark of vitality—an agent which, in many cases of disease, at an almost hopeless stage, will rally the shattered and almost dissipated vital forces, concentrate and generalize their action, and re-establish that series of atomic changes upon which vital action depends.

Cantharides has been used for a long time chiefly as a vesicatory, and for a few specialties, such as the treatment of leucorrhœa and incontinence of urine, some hopeless cases of dropsy, involuntary seminal emission, etc.; but I am not aware that it has ever been used in every-day practice, to accomplish those changes of pathological condition which we every day seek to effect by alteratives, tonics, stimulants, and vesicatories.

Old authors, and perhaps some recent ones, have spoken of the "blistering stage" of disease, and insisted on it as something definite; and I have no doubt many have understood it practically; but it does not appear to have ever become generally understood so clearly as to be matter of every-day observation in practice. Neither has the *modus operandi* of the action of a blister ever been, so far as I have read, clearly explained. Dr. R. Trask, of Strongville, O., a shrewd practitioner of much experience, remarked to me, several years ago, that he had no recollection of any case doing badly where blistering produced strangury. Since that time my observation, which has been considerable, justifies his remark, that strangury in blistering is a good symptom. What shall we say, then, to those writers who have proposed means to prevent this effect of epipastics?

In the autumn of 1854, typhoid fever and common continued fever prevailed to considerable extent in some of the Northern portions of Ohio. During that season I was called in frequently to see patients in whom congestion of the lungs had supervened at an advanced stage of the fever. In those cases, where any hopes from remedial agents remained, I ordered the application of emplast. canthar., with the view of producing revulsive and derivative action. The success was excellent in many cases; and it was at this time I made what has since appeared to me an important observation, which was this: In those cases which were at all remediable, the abatement of bad symptoms began within 30 minutes after the application of the plasters. When I first observed this in a single case, I naturally suspected it might be a coincidence; but further observation satisfied me that the same resulted in every case where blistering was imperatively demanded, and where the amount of plaster applied was large. Now, what did this prove? Certainly not that vesication was the cause of the improvement or change of pathological condition, for vesication with the best of plaster seldom occurs under four hours in adults,—even redness is not induced in much less time than three hours. Then it must prove that the effect either results from the plaster acting as a poultice to warm and shield the air from a part, or from absorption of the ingredients of the plaster. And what ingredient would more probably produce the effect than the cantharides?

The hint was sufficient, and I resolved to try the effect of cantharides administered generally, instead of topically, in certain cases which might present themselves. However, I did not put this resolution in practice till more than two years afterwards; for I was in poor health and much out of practice for that length of time. I will mention now a few cases from memory, as they occur to me.

The first case in which I made the trial direct was a feeble woman, aged about 50. She at the time had had obscure remittent fever nearly two months, which had resisted all remedies; she was quite delicate, and could endure but little medicine of any kind; she had enlarged spleen, and every few days had a "sinking" chill, each one reduced her lower than the preceding. On the occasion of the last recurrence of chill, which really prostrated her very low, I was called in during the chill. Pulse small, frequent, and feeble. Gave M. xl. cinct. cantharides (this for her was a large dose, for she could not endure much medicine of any kind generally.) In about 15 minutes she was quite rallied. Improved rapidly, and recovered from that day, without another chill.

Capt. J. P., aged 54, slim man, who had used much mercury years before. He now followed the trade of a tinner; troubled much with constipation and numbness of one leg. On the —— day of October, felt chilly about 10 o'clock; about the same hour, received news of the death of a son, absent from home; about noon, was seized with unconsciousness, and a persistent loquacity or jabbering, in which condition I found him about 1 o'clock P.M. He lay on the bed without moving hands or feet, face red, eyes natural, pulse moderately frequent, and constantly talking in a natural tone, but forming no complete words, and quite unconscious of all that was said to him. It was not clear whether this was the paroxysm of an intermittent, or a shock to the nervous system, occasioned by the sad intelligence, or both; but whether either or both, it seemed to bode evil to an individual who had for a long time had partial palsy and constipation. Ordered: ℞ Tinct. cantharides, aqua camph., aa fl℥ j. M. Dose, three drachms at once, and repeat two drachms every hour.

After he had taken the third dose, the anomalous symptoms entirely abated, and he took no more of the medicine. In the evening, gave a dose of camp. and carb. ammoniæ, after which he vomited freely; slept good; and next day was quite well; and continued as well as usual thereafter. There was no strangury in either of these cases.

In February, 1859, was called to see a boy aged 15; had been ailing several days; seemed unconscious, or at least to have no volition or speech; tongue white and slimy, pupils dilated, pulse irregular, from 80 to 90; voided scantily heavy urine, took no food or drink, and submitted passively to whatever was done to or for him. Practiced scarifying and cupping, on this and the following day, to the nape of the neck. Prescription: ℞ Tinct. cantharides, q. s. Dose M. xl. every four hours. Also, ℞ Iod. potass., grs. iv.—this dose very four hours: the two medicines to be given every two hours alternately. These doses were continued four days, though on the fourth the dose of cantharides was reduced to about 25 drops. The urine on the second day was copious and dark, and continued so, gradually growing of a lighter quality, till on the fifth it was full light, healthy color, when the cantharides was discontinued entirely. The boy was now quite rational, pupils natural, pulse regular—72. In this case there was very slight sensation with urinating.

I now refer the readers of the *Lancet* to a case mentioned in an article contributed by myself, entitled "Quinine in Pneumouia," and published in the August number of 1861, in which three large fly plasters were applied at once, and a fluid drachm of tinct. cantharides administered at the same time, and repeated in a forty-minim dose an hour afterwards, and half-drachm doses continued thereafter, at longer intervals,—all this with the happiest results, in an extreme state of typhoid pneumonia. There was no strangury in this case.

In a case of gangrenous erysipelas, where the life seemed to hang upon a thread, as the lungs were becoming engorged, a fluid drachm was given, and half-drachm doses continued for four days thereafter, with happy results, and no strangury. Other medicines were given in this case, such as quinine and tinct. ferri chlorid.; but no medicine made the visible impression at every dose as did the tinct. cantharides.

A case of animal poisoning in the month of August, 1861: a stout German laborer, aged about 40, had skinned a cow, which had died of some kind of epidemic, about five days previous to my visit. Felt poorly the next day after the skinning, and on the third day was quite unwell, and employed a disciple of Hahnemann, who attended him up to the time of my being called. Both arms were swollen and doughy, with numerous large, hard and prominent bluish vesications, from the size of a dime-piece to that of the palm of the hand nearly, dispersed on the fingers and arms, and the axillary glands swollen very much. There was at this time an anxions expression of countenance and difficulty of breathing, the skin dusky, and the rales in the lungs indicated a rapidly maturing congestion. It did not appear in this case that the specific (as I have sometimes thought it to be) for animal poison—caustic ammonia—would be sufficient to ward off the termination which appeared so imminent; the case demanded some remedy to act at once on the almost paralyzed capillaries, to restore their tone and rapidly eliminate effete matter. And what should that remedy be to turn the tide of life at this critical moment? Cantharides? Yes. I gave the tincture in fl℥ iss. doses, in conjunction with a suitable quantity of aqua ammoniæ. This quantity of cantharides was not repeated more than three times in the space of three or four hours. It was afterwards given in smaller doses and at longer intervals; quinine was also given after the full action of the cantharides was effected. I never saw what appeared to be a fatal tendency of disease more quickly and completely averted. I mean to say, the rapidly progressing congestion and death by sedative action were averted, and a case which appeared hopeless transformed to a hopeful one. Recovery was rapid under the use of ammonia and quinine, and iodine locally. There was no strangury in this case.

Now, some may say it was the caustic ammonia that did the business in this case. I have no doubt that caustic ammonia did as much in this case as it will in any case where there is so much to be done; but I by no means believe that the few drops of that medicine revolutionized the tendency of the accumulated evils, for the evolution was so precisely like what I have seen result from the administration of cantharides in so many instances.

—The following case will illustrate the anodyne or sedative effect of cantharides:—

Dr. R. Trask, of Strongsville, Ohio, to whom I had communicated my views of cantharides, was called in the night, about the 1st of last May, to see a young woman, about 16 years old. This girl was quite reduced in strength by hard labor, and had her spirits prostrated by family reverses; she had had leucorrhœa badly. At this time her appearance was quite alarming, her respiration was 75 per minute, and the pulse very frequent, but not in proportion to the frequency of respiration. The Dr. considered the case hysterical, in which, I have no doubt, he was right, and gave her some of the usual remedies, and lay down and slept three hours; when, on rising, he found his patient quite unchanged; he then gave her tinct. canth. fl℥ j., and ordered M. xl. to be given in about two hours in case there was no improvement. When he saw her again, at the end of four hours, the frequency of respiration was reduced to below 50 per minute. The medicine was continued in a smaller dose for some days afterward, in conjunction with iron. There was no strangury until the diminished dose was continued, as is usually done in leucorrhœa.

In the summer of 1860, A. C., an Irish laborer, of spare habit of body, and rather fond of whisky, had been sick a week with bilious fever, and was comatose—medicine having produced but little effect upon him. A friend called to see him on very important business, and tried for half an hour to arouse his attention, but failed to elicit recognition, although the business was very important. I gave the patient at this stage, pulv. camph., grs. iv., tinct. canth. fl℥ j. In twenty minutes patient aroused without solicitation, recognized his friend, extended his hand and spoke to him, and was able to converse sensibly.

I might quote many more cases, but let these suffice. I have given it in large doses in low stages of typhoid fever, in engorgement of the lungs, and, as will be seen above, in congestion or dropsy of the brain; in spasmodic diseases, and in very atonic cases of intermittent. I have also given it in cholera, of which I treated some cases in 1860, or cases which were no way distinguishable from cholera. One of these cases particularly went through the severe stages of cholera, with the characteristic purging and vomiting, cramps and blue surface, with parboiled hands. In this extreme condition, I gave him largely of the tinct. canth., and no other active medicine. Of course, I applied external warmth and gave warm drinks. Improvement was speedy after the medicine. No strangury.*

The reader is, no doubt, impatient to ask, "Why does not strangury result?" I might ask in reply, "Why should it?" I know that in cases where its peculiar action is plainly and clearly indicated the large doses may be given without strangury, and with the most happy results. In cases where it is not indicated strangury will result from one fluid drachm, generally.

When is cantharides indicated in the large dose? It is indicated at that very stage of disease which authors have pointed out as the "blistering stage." This will be the indication for it generally, and if the stage is properly marked, it will seldom fail of doing good, which will generally be perceived in 15 or 20 minutes after the dose is taken.

I by no means wish it understood that I disparage blistering. I have no argument against the revulsive or derivative action of vesication. I have seen much good result from the counter-irritation of epispastics and sinapisms, It will be seen by the context that I have used both the external and internal application of the fly in urgent cases. But there are some extreme cases of disease in which, if we should wait for the absorption of the cantharadin through the skin, when vitality and blood have almost forsaken the surface, the patient would sink beyond our reach. It is in this condition of lost vitality of the surface that the internal large dose of the fly displays its power to the most advantage.

I have, on several occasions, during my service in the army, laid on large blister plasters, for the purpose of securing a large amount of absorption of the fly, when I had not at hand preparations for internal use. The result was similar, but slower; but in some cases where the skin had lost much of its function the absorption was too feeble to benefit.

It is also indicated in several other states of disease in which blistering has not generally been looked to as important, viz.: A patient laboring under a disease of the heart, by a mistake in a prescription, had taken mercury till he was profusely sali-

* I use the officinal tincture, and have it carefully prepared under my own inspection. I prefer this to the powder, as being more active.

vated, with immense swelling of the tongue and whole region of the mouth. Alarming prostration ensued, and death by syncope or anæsthesia seemed imminent; he was unable to speak, and insensible. Tinct. cantharides was given in drachm doses and repeated often; he soon rallied, and in a few hours (five or six) the swelling had disappeared, and the tissues were restored to the elasticity—by which I mean that the prostration was reversed and followed by a tonic condition. This was a striking illustration; for in the morning the man was a disfigured and dying mass, and before the evening his features were quite natural and but little swollen.

But there should be established a general indication for giving so potent a remedy, and I think the following is the true one:—

*When in atonic, asthenic or adynamic disease it is a desideratum, from whatever cause, to produce general or local capillary tonicity, the internal use of cantharides will be indicated, and in quantity proportioned to the urgency of the demand.*

As an easy rule for the indication of cantharides, it may be stated thus: When turpentine is indicated to produce general action, cantharides is also still more indicated, if the indication is urgent; but they should not both be given.

One fluid drachm will generally be sufficient for a dose, but I would not hesitate to double this quantity in extreme cases; but, of course, these large doses need not be often repeated. I have seldom given over fl℥ iss. at a dose, with repetition of smaller doses from one to two hours after. The effect will be manifest in 20 minutes or less from the time of administration. The repetition of such doses should not be trusted to other judgments than those of the prescriber; for it is too potent a medicine to trifle with, and no more should be given than sufficient to accomplish the object, which will be definitely manifest by the improvement of the general symptoms, and the lowered frequency of pulse.*

I have no doubt this medicine is as potent for mischief as for good, if given too largely, or in very large doses on improper or false indications. In one respect it is like mercury—it will not fail to accomplish something, given in however large or small a dose. I have suggested that it acts by re-establishing the series of atomic changes upon which vital action depends; it is important that the atomic changes be not urged beyond a point of endurance, for then the tendency would be to destroy.

* I here advise whoever prescribes this medicine upon such indication as that pointed out above, to give the full dose, for it is the large dose, from forty minims to two drachms, that performs the great evolutions that I have described.

I have more than once seen life rekindled when the spark had almost gone out—when the Rubicon was passed; and the machinery kept in motion and reason upon her throne for many hours, and in a few cases for several days, by the action of this medicine, establishing an artificial life by continuing the atomic changes from which vital force is derived.

It appears to me that the cantharides acts primarily upon the capillaries by inducing tonicity, and in congestion diminishing their calibre, whereby the congestion is relieved; also vigorous absorption and secretion is promoted thereby. I am not certain whether there is any direct action of cantharides upon the kidneys. It appears that by the action of cantharides upon the general constituents of the entire organism a large amount of nitrogenized effete matter is thrown into the circulation which the kidneys rapidly elaborate into urine, whereby the quantity of dense urine is increased. This view is strengthened by the fact that as long as the urine continues to be dense or much colored, there will be no strangury, and no symptoms of irritation or excitement produced by the medicine; but when the urine becomes pale, the quantity grows less, and if the medicine is then continued, erethism results, also strangury. Hence we derive this rule: *Cantharides may be given in free doses* (*in cases where indicated*) *as long as the urine continues of a darker color than pale amber.*

I can not state how the urine is effeted, or how the kidneys are acted upon, in those cases where it is given purely as a diuretic in ascites and anascrca, not having had experience in prescribing it for such cases; but I am well convinced that if it could be borne in large dose in anasarca without exciting strangury, it would give a tonicity to the capillaries sufficient to discharge the fluids rapidly.

Besides this general action of cantharides, it seems to have a local action, as its effect in leucorrhœa, gleet, involuntary seminal emission and incontinence of urine is well known. But whether it really has this supposed local action, or operates by a general restoration of the capillary system, I am uncertain, notwithstanding I have successfully treated these various affections with it.

From the fact that strangury is not excited so long as the urine is copious and heavy, and that the quantity discharged is diminished after the color has disappeared—that is, after the proper effete atoms of the systems are discharged,—it appears not improbable that the irritation of the urinary passages is excited by crude and imperfectly decomposed atoms of the tissues of the body, or detritus which is not ready for elaboration into urine, brought in contact with their surfaces by the powerful decomposing action of the medicine. In confirmation of this view I submit the analogous fact, that the liver and the other glands become unduly excited after the full and proper action of mercury on the tissues and blood at large; and when there is not much nascent effete matter in the system these glands become excited the sooner.

Of its supposed aphrodisiac action I have seen no evidence whatever, notwithstanding I have watched closely for it. If it ever does excite any such action, it must be on persons laboring under no particular disease, or by a restoration of the lost tone in the worn out lecher. As it spends its force upon the effete atoms of the azotized constituents of the body, I can readily conceive the erethism which might be produced by it, in large doses, on an organism not having any such material for it to operate upon.

Now, in conclusion, I wish to say that I have written the foregoing facts because they are true, and ought to be known to the profession; and for the few conjectures submitted I ask your indulgence till they are further considered.—*Cincinnati Lancet and Observer.*

